# Nurses’ Experiences of Interprofessional Collaboration in Digitally Supported Hospital Discharge Planning: Qualitative Study

**DOI:** 10.2196/81961

**Published:** 2026-02-09

**Authors:** Mera Delima, Musheer A Aljaberi, Regidor III Dioso

**Affiliations:** 1 Faculty of Nursing, Lincoln University, Malaysia, Selangor, Malaysia; 2 Faculty of Health Sciences, Universitas Perintis Indonesia, Jl. Adinegoro, Simpang Kalumpang, Lubuk Buaya, Padang, West Sumatra, 25173, Indonesia, +62 813-6342-0560; 3 Franciscus Academy, Franciscus Gasthuis & Vlietland, Rotterdam, The Netherlands; 4 Research Centre Innovations in Care, Rotterdam University of Applied Sciences, Rotterdam, The Netherlands

**Keywords:** digital health, digital communication, discharge planning, electronic health records, health information systems, interprofessional collaboration, integrated patient progress notes, qualitative study

## Abstract

**Background:**

Effective interprofessional collaboration (IPC) in patient discharge planning is essential for ensuring continuity of care, improving patient outcomes, and strengthening coordination among health care professionals. Nurses often serve as primary coordinators due to their continuous engagement in patient care. However, the implementation of IPC continues to face barriers at the individual, team, and organizational levels. Many hospitals have adopted digital tools, such as integrated patient progress notes (IPPNs), to facilitate information sharing. Nevertheless, the use of these tools to support IPC remains suboptimal and has been insufficiently explored, particularly within the Indonesian digital health context.

**Objective:**

This study aimed to explore how IPPNs support IPC during patient discharge planning, particularly from the nursing perspective.

**Methods:**

A qualitative phenomenological study was conducted at a hospital in Bukittinggi, West Sumatra. Data were collected through in-depth interviews and a focus group discussion involving 9 purposively selected health care professionals. Thematic analysis was used to identify key patterns related to IPC practices and communication dynamics involving the use of IPPNs.

**Results:**

The findings revealed 3 main themes: (1) individual understanding and motivation in IPC, encompassing motivation, role expectations, personality style, and professional strengths; (2) team dynamics, including leadership, management, communication, and social support; and (3) organizational support for IPC, comprising collaborative culture, institutional goals, organizational structures, and the organizational environment. Participants perceived IPC as essential yet inefficiently utilized for coordinating patient care across disciplines, with limitations in standardization, accessibility, and clarity of digital documentation hindering effective collaboration.

**Conclusions:**

This study demonstrated that IPC practices were shaped by individual, team, and organizational factors, with digital communication holding a potentially transformative role in facilitating collaboration. These findings contribute to existing knowledge by highlighting context-specific challenges in Indonesian digital health settings, including digital literacy, system usability, and institutional support, which influence IPC and discharge planning outcomes. Integrating digital optimization within IPC frameworks may represent a valuable strategy for advancing digital health practices.

## Introduction

Patient discharge from hospital care represents a critical stage in the care continuum, particularly for individuals with chronic or complex conditions [1]. Effective discharge planning requires interprofessional collaboration (IPC) to ensure continuity of care and minimize adverse health outcomes [2]. Nurses play a central role as patient and family educators and function as key coordinators of interprofessional communication. However, IPC remains suboptimal in many health care systems, particularly in low- and middle-income countries (LMICs), contributing to prolonged hospital stays, medication errors, fragmented care, and reduced patient satisfaction [1,3].

Technological innovations have been identified as potential strategies for enhancing IPC. Web-based platforms, such as *Discharge Today*, have demonstrated improvements in team coordination without increasing workload, with most users perceiving the information as accurate and useful [3]. In situations with limited face-to-face interaction, such as shift handovers, these tools support continuity of information exchange among team members [4]. Virtual simulation–based interprofessional education (IPE) has also been shown to strengthen collaborative competencies among health care professionals, particularly nursing students [5]. However, the successful implementation of such interventions is highly dependent on health care professionals’ digital readiness, motivation, and technological competence.

Barriers to effective IPC persist at both institutional and individual levels. Organizational constraints, including weak leadership and inadequate managerial support, hinder technology-enabled collaboration and contribute to poor communication and reduced team performance [6,7]. At the individual level, resistance to change, heavy workload, limited digital literacy, and unclear professional roles impede collaboration, particularly during discharge transitions [2,6].

Digital documentation tools, including electronic health records, shared care plans, and standardized discharge forms, are designed to support IPC by improving clarity and consistency of clinical information [3]. However, practical challenges, including documentation fatigue, limited training, and system incompatibility, often limit their optimal use in clinical practice [4].

In Indonesia, hospitals have adopted an integrated patient progress notes (IPPNs), locally known as *Catatan Perkembangan Pasien Terpadu*, as part of the national electronic health records system to support multidisciplinary documentation [8]. Although nurses are required to routinely document patient progress using this system, studies have reported implementation inconsistencies, including incomplete entries, lack of standardization, and suboptimal use during discharge planning [9], compounded by unclear protocols, administrative workload, and limited digital literacy training [10].

Similar implementation gaps are evident in a type B teaching referral hospital in West Sumatra that has adopted the IPPNs system as part of its digital health strategy. However, despite being policy-mandated, no formal evaluation has examined the use of IPPNs in supporting IPC during discharge planning, particularly from nurses’ perspectives. Existing studies have primarily focused on technical or administrative aspects of documentation, with limited examination of IPPNs as a digital collaboration tool within clinical practice.

This hospital was selected as the study site because it functions as a regional referral center and teaching institution that actively implements national digital health integration and manages multidisciplinary, high-complexity cases requiring intensive interprofessional coordination. These characteristics make it representative of mid-level health care facilities transitioning toward integrated digital health systems. Therefore, this study aimed to explore how IPPNs are used as a digital collaboration tool to support IPC during patient discharge planning, with a particular focus on nurses’ perspectives and interprofessional practice.

## Methods

### Study Design

This study used a qualitative phenomenological design to explore health care professionals’ experiences of IPC in patient discharge planning supported by digital documentation in a hospital setting. The phenomenological approach was selected because it enables an in-depth exploration of participants’ subjective experiences and the meanings they ascribe to the phenomenon under investigation [11-15].

### Setting and Samples

This study was conducted between January and February 2025 at a type B teaching referral hospital in West Sumatra, Indonesia. The hospital has implemented IPPNs, locally known as *Catatan Perkembangan Pasien Terpadu*, as part of its digital service standards. The hospital was selected because it involves multiple health care professions in the collaborative process of patient discharge planning.

Participants were recruited using purposive sampling and comprised 9 participants: 3 physicians, 2 nurses, 2 pharmacists, and 2 nutritionists. This interprofessional composition was intended to reflect collaborative dynamics among health care team members. Data saturation was achieved, as no new themes emerged after the eighth interview, and the ninth interview confirmed thematic repetition. In phenomenological studies, data saturation is typically achieved with 6 to 10 participants who have direct experience with the phenomenon under investigation [12,13].

Although the study was nursing-oriented, 2 nurses were included because the primary focus was IPC in discharge planning rather than nursing practice alone. The selected nurses had direct clinical and coordinative experience in the discharge process, providing sufficiently rich insights to represent nursing perspectives. In phenomenological research, depth and richness of meaning are prioritized over sample size, making this sample appropriate for addressing the research objectives [13,14].

Participants were selected based on the following inclusion criteria: (1) Physicians must hold a valid practice license (Surat Izin Praktik, SIP) and a clinical competency certificate (Surat Keterangan Kewenangan Klinik, RKK), have at least 2 years of work experience at the hospital, and consent to participate; (2) Clinical nurses (level III) must possess an SIP and a signed RKK, have at least 2 years of work experience at the hospital, and consent to participate; (3) Pharmacists must hold an SIP and an RKK, have at least 2 years of work experience at the hospital, and consent to participate; and (4) Nutritionists must hold an SIP and an RKK, have at least 2 years of work experience at the hospital, and consent to participate.

All participants were anonymized and assigned codes to ensure confidentiality and protect personal information throughout the research process.

### Data Collection Procedures

Data were collected through in-depth interviews and face-to-face focus group discussions (FGDs) conducted at the hospital. The interview guide was developed based on the World Health Organization (WHO) IPC framework and King’s Interpersonal System Theory, focusing on 4 main domains: (1) perceptions of IPC in discharge planning; (2) roles and responsibilities of each professional group within the team; (3) barriers and facilitating factors influencing IPC implementation in the hospital; and (4) utilization of the IPPNs as a digital communication tool for coordination among professionals.

The following are the example interview questions:

1. How do you perceive IPC in the discharge planning process?
2. What challenges have you encountered when using IPPNs to coordinate with other professionals?
3. In your view, what factors could strengthen interprofessional teamwork in this hospital?

Each interview lasted approximately 45 to 60 minutes, was audio-recorded with participants’ consent, and was transcribed verbatim.

Following the individual interviews and initial thematic analysis, an FGD was conducted to validate and refine the themes that emerged from the interviews. The FGD served as a member-checking process to confirm the credibility of the findings and to provide cross-professional reflection on shared experiences. Importantly, the FGD was not intended to generate new themes but to enhance trustworthiness through data triangulation and ensure consistency of meaning across professional perspectives.

All participants received a clear explanation of the study objectives and procedures, and informed consent was obtained before data collection.

### Data Analysis

Data collection and analysis were conducted in 2 stages. The first stage involved 9 in-depth interviews using a semistructured interview guide consisting of open-ended questions based on the WHO framework for IPC and the core IPC competencies as outlined by the Institute of Medicine in the expert panel report. The information obtained from these face-to-face interviews was recorded with consent and transcribed verbatim. As the interviews and FGD were conducted in Bahasa Indonesia, data analysis was performed in the original language. Quotations included in the manuscript were translated into English carefully to retain conceptual accuracy. Interview data were analyzed thematically following the approaches proposed by Braun and Clarke [12,13], including open coding, theme development, and interpretation. The coding and theme categorization process was supported by NVivo 15 software (Lumivero) to ensure analytic consistency and traceability.

The reporting of this study adhered to the COREQ (Consolidated Criteria for Reporting Qualitative Research) checklist to ensure transparency and methodological rigor. The coding process followed the 6 phases of thematic analysis proposed by Braun and Clarke, including data familiarization, generation of initial codes, searching for themes, reviewing, defining, and naming themes. A preliminary codebook was developed inductively from the data, then refined through iterative discussions among the research team to ensure conceptual clarity. Coding discrepancies were resolved through peer debriefing and consensus meetings to achieve analytic consistency.

The second stage of data collection was an FGD conducted only once, including 4 participants selected from the initial participants based on the richness and relevance of the information provided during the previous analysis. FGD data were analyzed using the same thematic method, and the results from both stages were integrated using triangulation.

Potential bias was addressed in the FGD by ensuring equal opportunities for all participants to speak and by avoiding dominant voices that overshadowed others. The discussion was guided neutrally, with questions designed to elicit further reflection rather than primary responses.

### Trustworthiness and Rigor

To maintain the trustworthiness and rigor of qualitative data, the strategies outlined by Nowell et al [14,15] were applied as follows: credibility was ensured through data saturation, verbatim transcription, and triangulation of in-depth interviews and FGDs; transferability was supported by a detailed description of the study context, including participants’ profiles and data collection procedures; dependability was ensured through detailed documentation of the data collection and analysis processes; and confirmability was established through triangulation and discussions with study team members to ensure consistent and objective interpretations [14,15].

### Ethical Considerations

This study underwent ethical review and was approved by DR Drs M Hatta Brain Hospital, Bukittinggi, Indonesia (002555/KEP.RSOMH Bukittinggi/2024). Before data collection, participants were provided with a study information sheet explaining the purpose, benefits, methods, and participant rights. Participation was voluntary, and participants could withdraw at any time without consequences. Written consent was obtained from each participant after they read and understood the information provided. Confidentiality and anonymity were maintained by excluding individual identities from the report. Data were used solely for this study and stored securely in accordance with privacy policies. The research was conducted in accordance with the Helsinki Declaration. Participants did not receive any financial or material compensation for their participation in this study.

## Results

### Descriptive Results

Before presenting the main findings, an overview of participant characteristics is provided in Table 1, which summarizes the characteristics of 9 participants, all within the productive age range (25‐64 y) as defined by the WHO. Most participants were female (n=7, 78%) and held a master’s degree (n=7, 78%). The majority were married (n=7, 78%) and had more than 10 years of work experience (n=6, 66.7%).

**Table 1.** Participants’ characteristics.

Characteristics	Frequency, n (%)
Age (y)	
25‐64: adult (productive age range) according to WHO^a^	9 (100)
Sex	
Male	2 (22)
Female	7 (78)
Education	
Bachelor's degree	2 (22)
Master's degree	7 (78)
Marital status	
Married	7 (78)
Not married	2 (22)
Profession	
Doctor	3 (33.3)
Nurse	2 (22.2)
Pharmacist	2 (22.2)
Nutritionist	2 (22.2)
Work period (y)	
<5	0 (0)
5‐10	3 (33.3)
>10	6 (66.7)

a WHO: World Health Organization.

Participants represented 4 professional backgrounds: doctors, nurses, pharmacists, and nutritionists, reflecting multidisciplinary involvement in patient discharge planning.

### Health Care Professionals’ Experiences in Implementing IPC in Hospital Settings

#### Overview of Emergent Themes

Three main themes and 12 subthemes emerged from the analysis, describing health care professionals’ experiences in implementing IPC during patient discharge planning. The main themes and subthemes are summarized in Table 2.

**Table 2.** Themes of health professionals’ experiences in IPC^a^ practice at a type B teaching referral hospital in West Sumatra, Indonesia.

Code	Themes	Subthemes
1	Individual understanding and motivation in IPC	Motivation Role expectations Professional power Personality style
2	Team interaction dynamics in the discharge planning process	Group leadership Coping Communication Social support
3	Organizational support for IPC	Organizational culture Organizational goals Organizational domain The organizational environment

a IPC: interprofessional collaboration.

**Figure 1.**
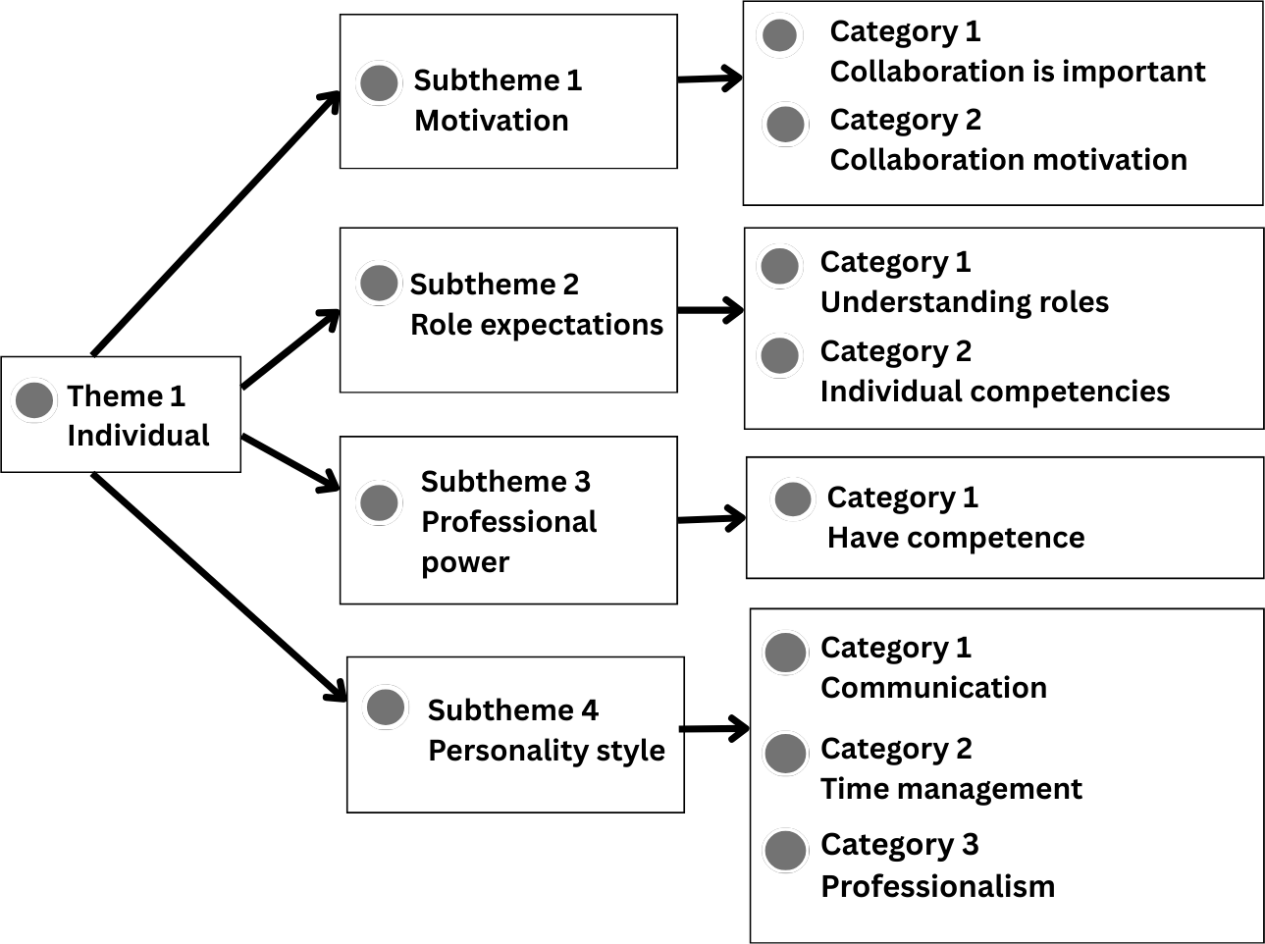
Thematic analysis of theme 1: individual.

**Figure 2.**
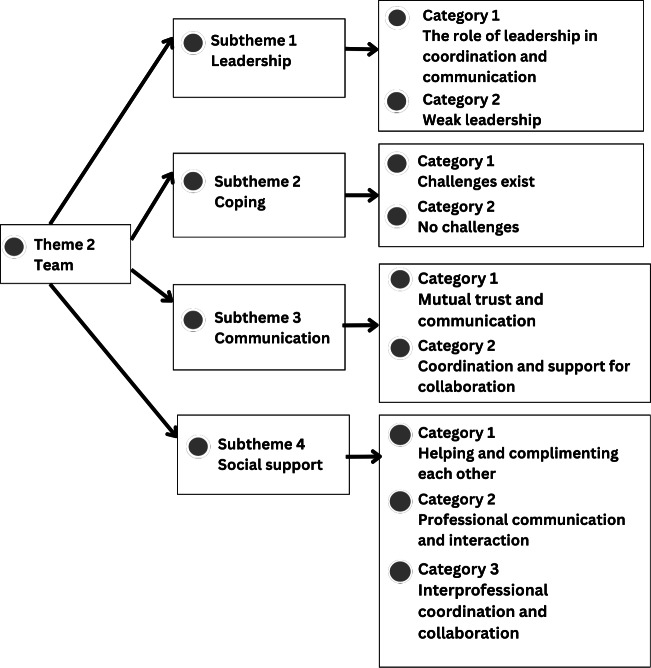
Thematic analysis of theme 2: team

**Figure 3.**
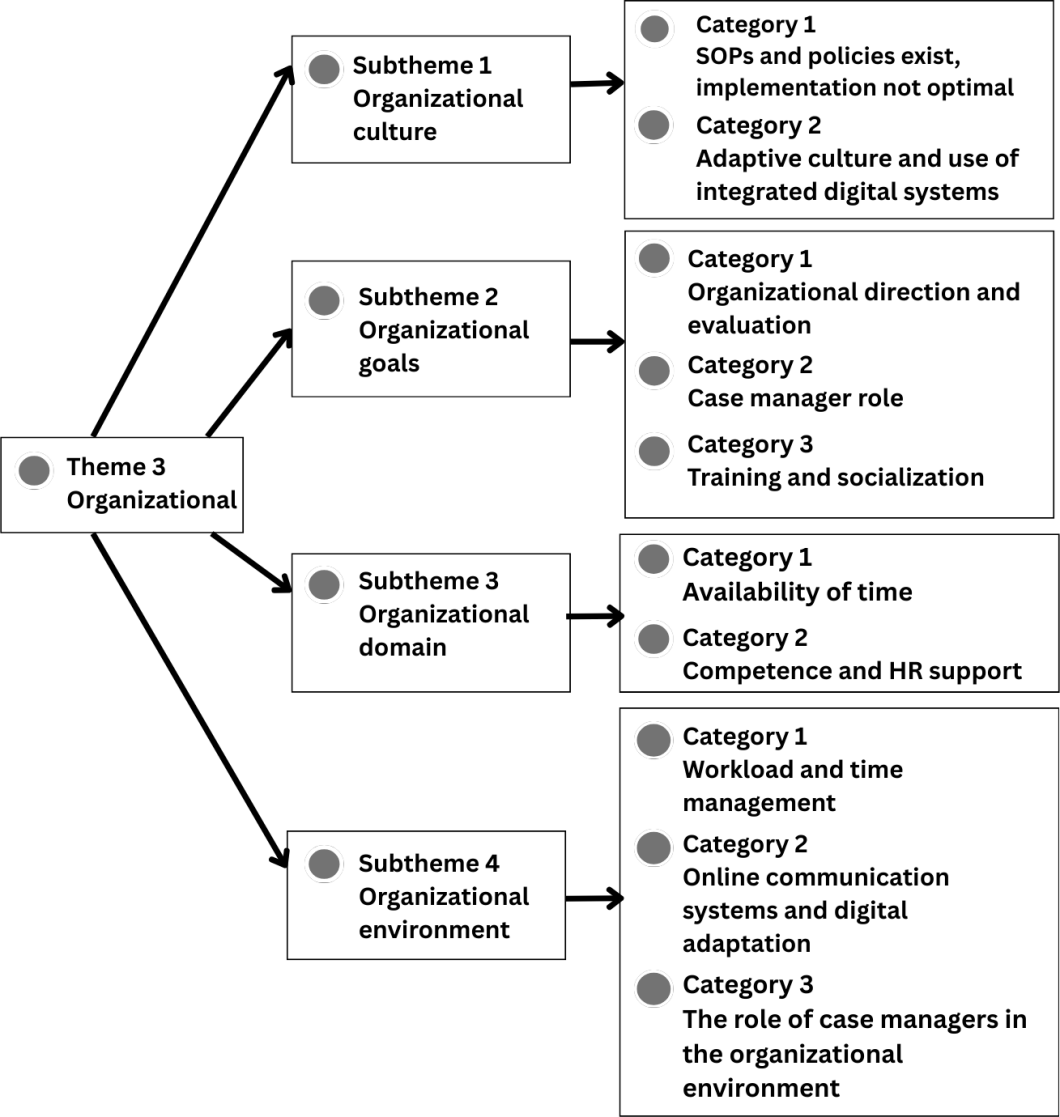
Thematic analysis of theme 3: organizational support. HR: human resource; SOP: standard operating procedure.

Further details of each theme, including supporting categories and illustrative participant quotations, are presented in Figures 1-3, which were generated using NVivo 15 to visualize the thematic structure, and described in the following sections.

#### Theme 1: Individual Understanding and Motivation in IPC

##### Overview of Theme 1

Qualitative findings suggest that individual factors play a crucial role in the implementation of IPC, particularly in the context of patient discharge planning. This theme comprises 4 subthemes: motivation, role expectations, professional authority, and personality styles, as presented in Figure 1.

Figure 1 illustrates theme 1, “individual factors”, which includes 4 subthemes with associated categories: (1) motivation, including the importance of collaboration and collaborative motivation; (2) role expectations, including understanding professional roles and individual competencies; (3) professional authority, including possession of professional competence; and (4) personality style, including communication, time management, and professionalism.

The following sections elaborate on each subtheme and present supporting quotations from the 9 participants.

##### Subtheme 1: Motivation

Internal motivation emerged as a key driver encouraging nurses to participate actively in collaborative discharge planning. Several participants recognized teamwork as essential to ensuring patient safety and continuity of care.

*Collaboration is essential, especially in planning patient discharge to avoid mistakes.*

[Participant 2]

Nurses also expressed motivation stemming from a desire to provide comprehensive patient education before discharge.

*I’m driven to collaborate so patients receive the right information before going home.*

[Participant 4]

This intrinsic drive was reinforced by shared professional goals and a sense of collective responsibility.

*I feel more enthusiastic when working with a cross-professional team because we share the same goal—the patient’s recovery.*

[Participant 5].

Some participants noted that motivation diminished when their contributions were not equally recognized.

##### Subtheme 2: Role Expectations

Role clarity influenced participation in discharge planning. Participants emphasized that understanding their professional responsibilities guided their involvement in team discussions.

*I understand that as a nurse, my responsibility is to educate the patient before discharge.*

[Participant 2]

Several participants identified interprofessional communication as part of their professional role.

*I report the patient’s condition to the team before they are discharged.*

[Participant 3]

Some nurses reported frustration when their roles were perceived as limited to routine tasks.

*Sometimes we’re seen only as medication providers, but we also educate patients about side effects.*

[Participant 9]

This perception gap between professions can act as both a facilitator and a barrier to collaboration, suggesting the need for clearer role delineation and mutual respect in team settings.

##### Subtheme 3: Professional Power

Professional hierarchy influenced collaborative dynamics. Some nurses expressed confidence in their clinical competence and independence.

*As a nurse, I feel capable of carrying out discharge planning.*

[Participant 3]

Others reported discomfort when expressing opinions in the presence of physicians.

*Sometimes it’s difficult to share opinions because we’re seen as subordinates.*

[Participant 5]

Training and institutional support were described as contributing to professional confidence.

*After attending training, I felt more confident managing discharge planning.*

[Participant 6]

Conversely, some participants reported that dominant attitudes from certain professionals affected collaboration.

*Sometimes doctors act like they know everything, which makes it hard for others to express their views.*

[Participant 7]

These findings illustrate the dual nature of professional power; it can empower individuals through competence and training or hinder collaboration through entrenched hierarchies.

##### Subtheme 4: Personality Styles

Interpersonal communication and personal traits influenced teamwork. Participants associated effective collaboration with openness, confidence, and adaptability.

*I’m used to coordinating and listening to team members’ input.*

[Participant 7]

*I prefer being open if there’s a different view, I take it as constructive feedback.*

[Participant 8]

Adaptability was identified as helpful when working with professionals from different disciplines.

*I’m adaptable and used to working across different professions.*

[Participant 9]

Nevertheless, communication gaps were occasionally reported, especially when individuals lacked confidence or were hesitant to speak up. This subtheme underscores that successful IPC depends not only on technical skills but also on interpersonal awareness and flexibility.

### Summary of Theme 1

Individual factors influenced participation in IPC during discharge planning. Participants described motivation, role understanding, professional hierarchy, and personal communication styles as shaping their collaborative experiences.

### Theme 2: Team Interaction Dynamics in the Discharge Planning Process

#### Overview of Theme 2

Team interaction dynamics played an important role in the discharge planning process and influenced the implementation of IPC. Participation and coordination among different professional groups were described as central to daily collaborative practice. Participants from various professions, including doctors, nurses, pharmacists, and nutritionists, described diverse interaction patterns that shaped how collaboration was formed, maintained, or hindered during discharge planning. These interaction dynamics encompassed leadership, communication, coordination, and social relationships within the health care team. The identified patterns illustrate how team members worked together to support patient discharge planning in routine practice. The structure of the themes, subthemes, and categories related to team interaction dynamics is presented in Figure 2.

Figure 2 presents Theme 2, “team interaction dynamics”, which includes 4 subthemes with associated categories: (1) group leadership, including leadership roles in coordination and communication and weak leadership; (2) coping, including collectively managed challenges and absence of challenges; (3) communication, including mutual trust in team communication and coordination and support for collaboration; and (4) social support, including helping and complementing each other, professional communication and interaction, and interprofessional coordination and collaboration.

#### Subtheme 1: Leadership

Group leadership influenced the effectiveness of collaboration in discharge planning. Participants described leaders as facilitators who initiated meetings, allocated responsibilities, and guided discussions.

*Our leader always starts the discharge planning meeting and divides the tasks.*

[P1]

*The team leader always guides the discharge planning discussion firmly but openly.*

[P2]

Participants described leadership styles characterized by fairness and openness: “Our leader encourages all members to express their opinions” (P3), and “Our leader is wise and willing to listen to all input” (P5). Some participants highlighted emotional control and role modeling by leaders: “Our team leader can maintain harmony, not authoritarianism” (P6), and “Our leader sets an example and supports joint decisions” (P7). Collectively, these narratives underscore leadership that balances authority with inclusivity.

#### Subtheme 2: Coping/Handling

Participants described both the presence and absence of challenges in IPC. Reported barriers included inconsistent evaluation, limited family engagement, and uneven participation across professions: “The challenges exist, especially because there has been no proper evaluation” (P1); “Coordination is a challenge because not all professions are present during joint visits” (P5). Differences in patient contact among professionals were also noted (P3). Some participants reported minimal challenges due to clear standard operating procedures (SOPs) and defined roles: “There are no challenges because all professions already have clear SOPs” (P6).

#### Subtheme 3: Communication

Participants described communication as occurring primarily through digital platforms, particularly WhatsApp groups: “Communication is carried out via WA groups” (P2, P7, P8, P9). Verbal discussions and documentation were also used to share patient updates: “We routinely inform each other about patient progress” (P1); “I routinely report patient progress to the team via medical records” (P3). Some participants reported communication challenges among new staff unfamiliar with interprofessional communication practices: “There are minor challenges if there are new employees who do not yet understand the ethics or interprofessional communication” (P6). This indicates the need for structured orientation and communication training within multidisciplinary teams.

#### Subtheme 4: Social Support

Participants described social support as contributing to team cohesion. They reported mutual trust, respect, and shared responsibility. As participant 1 stated, “I feel supported by other team members, so I am more confident,” while participant 3 added, “We respect each other; no one feels more important.” Participants also described encouragement and practical support among team members: “My team members always give encouragement and moral support” (P4). Moreover, collective accountability was evident when colleagues substituted for absent members without complaint: “If someone is absent, the team still covers for each other without protesting” (P6); “If someone is absent, the others are ready to change shifts without any problems” (P8).

### Summary of Theme 2

Team interaction dynamics influenced IPC during discharge planning. Participants described leadership, communication, coping with challenges, and social support as shaping their collaborative experiences.

### Theme 3: Organizational Support for IPC

#### Overview of Theme 3

Organizational support influenced the implementation of IPC in patient discharge planning. Participants described the role of policies, management, resources, information systems, and work culture in shaping collaborative practice. The structure of the themes, subthemes, and categories for organizational support is presented in Figure 3.

Figure 3 presents Theme 3, “organizational support”, consisting of 4 subthemes with associated categories: (1) organizational culture, including existing SOPs and policies with suboptimal implementation and adaptive culture through integrated digital systems; (2) organizational goals, including organizational direction and evaluation, case manager roles, and training and socialization; (3) organizational structure, including availability of time and competence supported by human resources; and (4) organizational environment, including workload coverage, time management, online communication systems, and the role of case managers in supporting IPC.

#### Subtheme 1: Organizational Culture

Participants reported the presence of SOPs and policies supporting discharge planning. However, limited dissemination and inconsistent implementation were noted.

*There are SOPs, but access is uneven across units, which can hinder implementation.*

[Participant 4]

*[A]lthough collaboration is mandated, the execution remains suboptimal.*

[Participant 9]

Some participants described the integration of electronic medical records as supporting collaboration. For example, a participant shared, “The new H-1 discharge policy is linked to electronic medical records, making it more transparent” (Participants 3, 5).

Others noted that implementation at the unit level remained inconsistent: “Although management has established procedures, their execution at the ward level still needs improvement” (Participants 2, 5). A formal structure supporting IPC exists, while cultivating a shared culture that translates into consistent, collaborative behavior in daily clinical work remains a challenge.

#### Subtheme 2: Organizational Goals

Participants described management support for collaboration through direction, case management, training, and evaluation.

*There are directions from management, but no overarching policy; however, patient satisfaction remains a central focus.*

[Participant 2]

*The presence of a case manager facilitates cross-professional decisions.* [Participant 6]

*Periodic training and evaluation are carried out to adjust discharge planning practices.*

[Participant 8]

Some participants highlighted the role of digital systems in monitoring collaboration:

*The iKame system allows us to track multidisciplinary participation.*

[Participant 7]

#### Subtheme 3: Organizational Structure

Participants reported that the availability of discharge planning teams and digital tools supported collaboration. Several participants described sufficient time allocation and improved efficiency through electronic systems. A participant shared, “Time is sufficient as long as roles are clear” (Participant 4), while another added, “Using the electronic system saves time compared to manual charting” (Participant 9). However, human resource limitations were also reported: “Time remains a challenge, but we hope things improve with more staff” (Participants 3, 5).

Facilities and digital access were described as adequate: “computers are available” and “nutrition leaflets and counseling are provided” (Participant 2). Participants also described the hospital’s electronic system as structured and role-based: “the IT system supports collaboration” and “access is structured based on role and profession” (Participants 6, 7). In summary, structural elements such as time, facilities, and information systems play a critical role in enabling IPC, although workload distribution and continuity of IPE still require improvement.

#### Subtheme 4: Organizational Environment

Participants generally perceived the work environment as supportive of collaboration: “The work environment supports collaboration and discussion” (Participant 2). Scheduling and coordination challenges were commonly reported. For example, “lack of adherence to schedules affects plans like physiotherapy” and “conflicting rounds and outpatient duties disrupt communication” (Participant 6).

Some participants suggested integrated scheduling and dedicated interprofessional meetings. “More integrated scheduling and dedicated interprofessional meeting time” (Participant 8). While digital systems were viewed as helpful, some participants preferred direct communication: “IT helps, but direct coordination is still more effective” (Participant 7).

Participants also mentioned the need for regular evaluations: “There should be routine meetings and comprehensive evaluations beyond administrative aspects” (Participant 2). Importantly, individual commitment was stated: “Even with full organizational support, without individual commitment, collaboration won’t work” (Participant 9).

Few participants consider IPC to be running optimally. However, the results suggest that organizational improvements, particularly in scheduling, evaluation, and communication, are still needed to sustain collaborative efforts.

### Summary of Theme 3

Participants described organizational policies, digital systems, managerial support, training, case managers, and work environment as influencing IPC during discharge planning. They also reported challenges related to policy implementation, scheduling, workload, and communication.

## Discussion

### Principal Findings

The findings of this study indicate that the success of IPC in patient discharge planning is influenced by 3 main factors: individual factors, team dynamics, and organizational support. Individual factors include motivation, role understanding, professional competence, and personality styles; team dynamics involve leadership, communication, problem-solving abilities, and social support; and organizational support encompasses work culture, service goals, resource availability, and a supportive work environment. These findings underscore that IPC effectiveness depends on the interaction between personal capabilities, effective team processes, and organizational structures.

Before discussing the findings in detail, participant characteristics should be considered, as they influence experiences and perceptions of IPC. All participants (n=9) were within the productive age range of 25 to 64 years, reflecting readiness for IPC and adaptation to digital systems [16]. Most participants were female (78.0%), consistent with the gender distribution in nursing and public health professions, which may influence collaboration dynamics through communicative and empathetic approaches [17]. Most participants held a master’s degree (78%), indicating strong academic capacity for understanding IPC concepts and applying digital communication in clinical practice [18,19]. The majority had more than 10 years of work experience (66.7%), contributing to professional maturity and confidence in interprofessional communication [17].

Although only 2 nurses participated, they were selected due to their strategic role as primary coordinators of discharge planning, making them representative of nursing competencies in IPC [16].

### Exploring the Professional Experiences of Nursing Professionals

#### Theme 1: Individual Understanding and Motivation in IPC

##### Overview of Individual Factors Influencing IPC

The findings demonstrate the critical role of individual factors in IPC implementation during discharge planning. Motivation, role expectations, professional power, and personality traits shaped health care professionals’ engagement and readiness. These findings align with international IPC literature, highlighting individual awareness and intrinsic motivation as foundational to effective collaboration.

##### Motivation

Motivation emerged as a strong internal driver rooted in responsibility for patient safety and care continuity. This aligns with Smith et al [5], who showed that interprofessional simulation strengthened motivation and collaborative readiness, and with Reinders et al [20], who emphasized intrinsic motivation in sustaining discharge coordination. In Indonesian settings, motivation is influenced by workload, resource constraints, and institutional incentives, indicating that it is both intrinsic and contextually shaped [20].

This study further shows that digital platforms such as IPPNs and WhatsApp function as external motivators by facilitating timely information exchange and shared accountability, consistent with global evidence on digital engagement in teamwork [16]. However, infrastructure limitations, technical barriers, and workload concerns remain as challenges [21]. Thus, technology-supported motivation requires organizational training, leadership, and incentives, particularly in LMIC contexts where motivation is more relational than structurally reinforced.

##### Role Expectations

Clear role understanding enhanced IPC participation, especially in patient education and communication. Conversely, undervalued or ambiguous roles—particularly among nurses—limited engagement. These findings support Tong et al [22], who found that role clarity improves collaboration and mutual respect. In Indonesia, hierarchical traditions and inconsistent policy enforcement continue to constrain role clarity [9,10]. Compared with high-income settings where roles are codified through standardized policies and digital protocols, local adaptation of IPC policies remains essential [16].

##### Professional Power

Professional hierarchy remained a persistent barrier. Despite clinical competence, some nurses hesitated to voice opinions due to perceived lower status. This mirrors Tan et al [23] and Nie et al [4], who reported that digital tools alone do not flatten hierarchies without inclusive leadership and policy support. In LMIC contexts, unequal authority and weak institutional enforcement limit the transformative impact of digitalization on power relations [4].

##### Personality Style

Personality traits such as openness, adaptability, and empathy facilitated IPC, consistent with prior studies linking emotional intelligence to effective handoffs and reduced information loss [10,24,25]. In the Indonesian context, strong family involvement added complexity to discharge planning, requiring sensitivity to sociocultural dynamics [26]. Comparable findings in high-income settings also highlight emotional intelligence as a key IPC enabler [27]. These results indicate that IPC effectiveness is shaped not only by systems but also by personal and interpersonal competencies [28].

##### Critical Reflection

These findings reinforce that IPC is shaped by individual attitudes and professional culture in addition to formal systems. Digital platforms can enhance coordination, but their effectiveness depends on leadership, organizational readiness, and equitable governance. Persistent hierarchies and uneven policy implementation in LMICs underscore the need for capacity building that integrates reflective leadership, interprofessional mentoring, and psychological safety.

### Theme 2: Team Dynamics in IPC for Discharge Planning

#### Overview of Team Interaction Dynamics

Team dynamics revealed the importance of leadership, communication, coping strategies, and social support. While digital platforms facilitated coordination, collaboration quality ultimately depended on leadership consistency, institutional accountability, and cultural readiness.

#### Group Leadership and Participatory Team Culture

Inclusive leadership fostered trust, shared accountability, and psychological safety, consistent with Bornman and Louw [29] and Keniston et al [30]. Conversely, inconsistent leadership resulted in fragmented communication and unclear roles, reflecting uneven policy implementation and limited leadership development common in LMICs [5].

#### Coping and Operational Challenges in Coordination

Teams faced logistical challenges such as inconsistent participation and fragmented workflows, echoing findings by Nie et al [4]. Clear SOPs and predefined roles mitigated some challenges, aligning with Buljac-Samardzic et al [31]. Persistent hierarchies and uneven enforcement reflected deeper governance barriers, while family involvement occasionally delayed decision-making when authority was unclear [31].

#### Communication Infrastructure and Team Transparency

Hybrid communication using WhatsApp, face-to-face meetings, and documentation supported coordination, consistent with Keniston et al [30] and Teuwen et al [32]. However, message overload and unequal participation persisted without shared digital literacy and leadership oversight [21,33-35]. Compared with high-income settings, IPC in Indonesia remains dependent on interpersonal initiative rather than integrated systems [27,34]

#### Social Support and Emotional Climate

Mutual respect, encouragement, and role flexibility strengthened team resilience, consistent with Cadel et al [34]. These findings align with evidence from high-income contexts showing emotional cohesion as a universal driver of IPC effectiveness [35-42].

#### Critical Reflection

Digitalization facilitates coordination but does not eliminate hierarchies or policy inconsistencies. Compared with high-income settings, IPC success in Indonesia relies more heavily on interpersonal leadership and local initiative, highlighting the need for aligned policy, leadership development, and collaborative governance.

### Theme 3: Organizational Support for IPC

#### Organizational-Level Support for IPC

Organizational support emerged as a prerequisite for sustainable IPC, encompassing culture, goals, structure, and work environment.

#### Organizational Culture

Although SOPs and policies exist, uneven dissemination and weak supervision limited consistent implementation. Hierarchical norms continued to shape communication despite digital tools. These findings align with Redzewsky et al [40] and Ishii et al [41], emphasizing that policy without localized integration fails to sustain IPC.

#### Organizational Goals

Leadership support through case managers, training, and evaluation facilitated IPC, although the absence of a comprehensive IPC policy remained a barrier. These findings align with Labrague et al [42], who linked supportive environments to improved safety outcomes.

#### Organizational Domain

Structural supports such as discharge teams, digital systems, and facilities enhanced IPC efficiency [43-46]. However, workload imbalance and limited IPE persisted [45]. Rawlinson et al [46] emphasized that the success of IPC was strongly influenced by team competencies, including effective communication, clearly defined roles, and consistent organizational support.

Adopting digital systems, such as electronic medical records and the iKame app, streamlines coordination and reduces time burden across professions. However, human resource shortages and compressed work schedules continue to present major barriers. Buljac-Samardzic et al [31] suggested that team structure optimization, including task clarity, adherence to SOPs, and structured time allocation, could alleviate operational barriers to IPC. Digital platforms such as IPPNs and WhatsApp improved responsiveness but remained informally governed, limiting institutional monitoring.

#### The Organizational Environment

The organizational environment plays a critical role in enabling effective IPC. Factors such as the availability of resources, dedicated time allocation, and structured workflows are essential for fostering a productive collaborative climate. Although digital technologies have been introduced to support interprofessional communication, several structural barriers persist, including noncompliance with schedules, overlapping clinical responsibilities, workload pressure, and a lack of integrated cross-professional scheduling systems. Ginting et al [47] emphasized that while digital tools could facilitate IPC, their effectiveness was limited in the absence of a supportive organizational culture and inclusive leadership.

Beyond systemic factors, individual commitment remains essential to ensure sustainable collaboration. A previous study found that interprofessional trust emerges from the quality and regularity of relationships, individual attitudes, and a supportive organizational culture, and that trust, in turn, enhances communication, coordination, teamwork, and ultimately collaborative outcomes [48]. Similarly, Reeves et al [49] showed that workplaces promoting team reflection and shared evaluation cultivated trust and accountability among professionals.

When compared to high-income settings, where IPC practices are often embedded in digitalized and standardized systems, hospitals in Indonesia face persistent challenges related to uneven digital infrastructure, informal communication channels, and resource limitations. Similar barriers have been observed in other low- and middle-income contexts such as Ethiopia and the Philippines, where digital and managerial gaps hinder consistent IPC outcomes. These contrasts highlight that while policy frameworks may be globally aligned, contextual and resource-based differences shape IPC implementation in diverse health systems [50-54].

These inconsistencies often stem from institutional hierarchies, variable managerial commitment, and uneven digital literacy among professionals, which prevent the full translation of collaborative policies into daily clinical practice [55].

### Holistic Interdependence Between IPC and Organizational Support

Cadel et al [34] and Smith et al [56] reported that peer substitution and team solidarity were critical to sustaining IPC, particularly during workforce fluctuations. Interprofessional simulation-based learning was also shown to strengthen team relationships and preparedness for role substitution when necessary. These findings emphasize the importance of organizational support mechanisms, including structured training and adaptive team models that can withstand staffing dynamics.

### Link to Regional Evidence

A systematic review found that satisfaction with organizational support and positive attitudes toward IPC were associated with collaboration effectiveness, although not often statistically significant [50]. This highlights the need for systemic approaches to improve working conditions, promote regular training, and implement inclusive interprofessional models in clinical practice.

### Critical Reflection

Organizational support for IPC depends on alignment between structure, culture, and leadership. Policies and digital tools alone are insufficient without consistent supervision, organizational learning, and reflective practice. Strong leadership commitment and structured evaluation forums are essential to embed IPC as a sustainable norm.

### Strengths and Limitations

This study’s strengths include its qualitative phenomenological approach, inclusion of multiple professions, and focus on digital documentation (IPPNs) within a middle-income country context. Methodological rigor was ensured through triangulation and trustworthiness criteria, contributing to theory building in IPC within resource-constrained systems.

Limitations include the single-site setting and small sample size. The study did not fully examine system usability or interoperability, and cultural hierarchies may limit transferability. Future research should adopt multisite and mixed-method designs to explore system-level and leadership influences on IPC outcomes.

### Conclusion

In conclusion, this study revealed the complex dynamics that underlie IPC in hospital discharge planning. A total of 3 interrelated themes emerged: individual understanding and motivation, team interaction dynamics, and organizational support for IPC. The findings demonstrated that intrinsic motivation and clear role expectations were fundamental in fostering individual readiness for collaboration. IPE played a significant role in strengthening professional identity and enhancing collaborative competence among nursing professionals.

Professional hierarchies and leadership styles influenced collaboration outcomes. Power imbalances hindered open communication and participation, whereas inclusive leadership fostered psychological safety and active engagement. Organizational support encompassing culture, structure, and systems proved essential, although challenges persisted in coordination, communication, and policy implementation.

Effective IPC in discharge planning requires a holistic approach that integrates individual preparedness, cohesive team dynamics, and strong organizational commitment. Actionable strategies, such as structured interprofessional orientation programs, regular digital literacy training, and consistent policy dissemination at the unit level, could strengthen collaborative practice and ensure its sustainability. These efforts can promote more integrated, patient-centered care and enhance continuity of care post discharge.

The findings provide implications for policymakers, hospital administrators, and nursing education institutions to incorporate IPC principles into professional development programs, digital health initiatives, and institutional governance frameworks, supporting collaboration as a sustained norm within health care systems.

## Abbreviations

COREQ: Consolidated Criteria for Reporting Qualitative Research
FGD: focus group discussion
IPC: interprofessional collaboration
IPE: interprofessional education
IPPN: integrated patient progress notes
RKK: Surat Keterangan Kewenangan Klinik
SIP: Surat Izin Praktik
SOP: standard operating procedure
WHO: World Health Organization
